# Stroma‐and Tumor‐Associated Predictive Features in Salivary Gland Adenoid Cystic Carcinoma of the Head and Neck

**DOI:** 10.1111/jop.13589

**Published:** 2024-11-10

**Authors:** Aleksi Rytkönen, Hanna K. Laine, Antti Mäkitie, Caj Haglund, Jaana Hagström, Alhadi Almangush, Ilmo Leivo

**Affiliations:** ^1^ Department of Pathology Oulu University Hospital Oulu Finland; ^2^ Department of Oral Pathology and Radiology University of Turku and Turku University Hospital Turku Finland; ^3^ Department of Oral and Maxillofacial Diseases University of Helsinki Helsinki Finland; ^4^ Department of Otorhinolaryngology—Head and Neck Surgery University of Helsinki and Helsinki University Hospital Helsinki Finland; ^5^ Division of Ear, Nose and Throat Diseases, Department of Clinical Sciences, Intervention and Technology Karolinska Institutet and Karolinska University Hospital Stockholm Sweden; ^6^ Research Program in Systems Oncology, Faculty of Medicine University of Helsinki Helsinki Finland; ^7^ Research Programs Unit, Translational Cancer Medicine Program University of Helsinki Helsinki Finland; ^8^ Department of Surgery University of Helsinki and Helsinki University Hospital Helsinki Finland; ^9^ Department of Pathology University of Helsinki and Helsinki University Hospital Helsinki Finland; ^10^ Institute of Biomedicine, Pathology, University of Turku and Turku University Central Hospital Turku Finland

**Keywords:** adenoid cystic carcinoma, survival, tumor budding, tumor‐infiltrating lymphocytes, tumor‐stroma ratio

## Abstract

**Background:**

There is lack of knowledge on the utility of prognostic histopathologic characteristics in adenoid cystic carcinoma (ACC) of the head and neck. We evaluated the prognostic value of tumor and stroma‐related histopathologic features in ACC.

**Materials and Methods:**

A total of 65 cases of ACC from minor and major salivary glands were included in this study. We evaluated tumor budding, tumor‐infiltrating lymphocytes (TILs), and tumor‐stroma ratio (TSR) in hematoxylin and eosin (HE) stained sections.

**Results:**

Stroma‐rich ACCs recurred more frequently (*p* = 0.029) during follow‐up and associated with distant metastasis (*p* = 0.038). In multivariable analysis, stroma‐rich tumors associated with poorer disease‐specific survival with a hazard ratio of 3.76 (95% CI 1.10–12.83, *p* = 0.034). ACCs commonly showed a low infiltration of TILs as 89% of the tumors was characterized by an immune desert pattern. Low infiltration of TILs associated significantly with increased tumor budding (*p* = 0.039).

**Conclusion:**

Adverse features of TSR and tumor budding are widely expressed in ACC, and stroma‐rich tumors are associated with poor prognosis. Low number of TILs in ACC tissue indicates a weak immune response by the host and illustrates the nature of ACC as a relentless malignancy.

## 1 Introduction

Adenoid cystic carcinoma (ACC), a rare neoplasm, is frequent among salivary gland malignancies [1]. ACC may present with late recurrences and metastases up to 30 years from the primary diagnosis causing significantly impaired survival [2]. Diagnostics and predicting the behavior of ACC can be challenging. In previous studies it has been shown that solid growth pattern and perineural invasion are aggressive predictive behavioral features of ACC [3].

Recently, Persson et al. have described potential prognostic markers in ACC such as tumor suppressor genes *TP73* and *PARK2* [4]. The utility of several tumor‐ and stroma‐related prognostic and predictive histopathologic characteristics have been identified recently in various cancers including head and neck cancers using hematoxylin and eosin (HE) staining [5, 6, 7, 8, 9]. Such characteristics include tumor budding, tumor‐infiltrating lymphocytes (TILs), and tumor‐stroma ratio (TSR), none of which have been studied in ACC so far.

Tumor buds are clusters of less than five tumor cells located at the invasive front of the main tumor mass [10]. High number of tumor budding has been reported to correlate with an aggressive disease and worse overall prognosis in oral and colorectal cancers, among others [10]. In salivary gland cancers including ACC, the tumor stroma has been less studied. Cell signalling by stromal cells, such as extracellular matrix fibroblasts and inflammatory cells has been shown to participate in behavioral regulation of malignant epithelial cells influencing tumor invasiveness and thereby patients' prognosis and clinical outcome [11]. Notably, stroma‐rich tumors appear to have worse prognosis [12]. On the other hand, TILs are specific inflammatory cells that infiltrate into cancer tissue. In cancer treatment, it might be beneficial to develop methods to stimulate host defense and thus increase the number of TILs [13]. In general, salivary gland cancers, including ACC, do not induce strong immune responses, as shown by their low numbers of TILs [14].

The aim of this study was to evaluate the prognostic value of tumor and stroma‐related histopathologic features in ACC including tumor budding, TSR, and TILs.

## 2 Materials and Methods

We studied retrospectively ACCs of minor and major salivary glands. The tumors were reclassified according to the WHO Classification of Head and Neck Tumors (2017) [3]. The data of this series have been reported in two of our earlier studies divided into minor and major salivary gland ACC series [15, 16]. Briefly, the patients were selected from the years 1974–2012 from the Helsinki University Hospital archives. All head and neck ACC cases with histological slides available for evaluation were included. The Helsinki University Hospital Research Ethics Board (HUS Regional Committee on Medical Research Ethics, Dnro 31/13/03/02/2010, 01 February 2010) approved the study design, which was retrospective with no effect on the treatment of patients. Patient consent was waived by the Finnish National Supervisory Authority for Welfare and Health (decision nro 425/05.01.00.06/2009 and 10 041/06.01.03.01/2012).

In the evaluation of histopathologic features, we used HE‐stained slides and followed previously described methods as follows: to assess tumor budding, a glass slide containing whole tumor tissue was evaluated at low magnification (×4 objective lens) and using a ×20 objective lens the area with the highest number of tumor buds was chosen for counting [7]. Tumors with less than 5 buds were scored as low budding and those with 5 buds or more were scored as high budding, as described previously [17]. TILs in stromal areas were assessed following the criteria of the International Immuno‐Oncology Biomarker Working Group [18, 19]. The whole tumor was evaluated at low magnification using a × 5 objective lens followed by higher magnification with a × 10 objective lens. Number of stromal TILs was defined as the percentage of stroma occupied by infiltrating lymphocytes. The average density of TILs was reported for each tumor. For quantitation of TSR, the recently introduced guidelines were followed [20, 21]. Samples were observed at a low magnification (×5 objective) to select the area with the highest amount of tumor‐associated stroma. Using a higher magnification (×10 objective), the percentage of tumor‐associated stroma was assessed in fields where cancer cells were present on all four sides. In case of tumor heterogeneity, the area of the highest stromal percentage was chosen as indicated by the guidelines [20, 21]. Two observers experienced in head and neck pathology (A.A. and I.L.) who were blinded to the clinicopathologic data assessed the samples following the above‐mentioned criteria.

Statistical analyses were conducted with SPSS software (version 27). Overall survival (OS) was defined as the period between the last day of treatment and the date of death or last follow‐up. Disease‐specific survival (DSS) was calculated as the period between the last day of treatment and death of disease. Disease‐free survival (DFS) was defined as the interval from the last day of treatment to recurrence (if any). Correlation between the clinicopathologic features and the evaluated biomarkers was analyzed by cross‐tabulation and the Pearson Chi‐Square test.

For Cox regression analyses, we reported a hazard ratio (HR) and 95% confidence interval (95% CI). Statistical significance was assumed at *p* value < 0.05. We conducted univariate analysis for each parameter. Then the multivariate Cox regression model was conducted to verify the prognostic independence when age, gender, growth pattern, and perineural invasion were adjusted. Each of the examined histopathologic features (i.e., TSR, TILs, and tumor budding) was entered into a multivariate model comprising the above‐mentioned factors (i.e., age, gender, growth pattern, and perineural invasion).

## 3 Results

We included all available 65 ACC cases from the Helsinki University Hospital archives. Of these, 39 (60.0%) were women and 26 (40.0%) men. Table 1 shows patient and tumor characteristics. Majority of the patients (*n* = 43, 66.2%) were ≤ 60 years. The mean age at the time of diagnosis was 54 years (range 24–80 years). A total of 27 (46.5%) cases were diagnosed at an advanced stage. In 63 (96.9%) cases the treatment was intended to be curative. Thirty‐three (50.8%) patients developed a recurrence, while 22 (33.8%) died of the disease. The mean follow‐up time was 7.8 years.

**TABLE 1:** Patient and tumor characteristics of 65 patients with adenoid cystic carcinoma.

Sex	Men	26 (40%)
	Women	39 (60%)
Age range	24–80 years, (median 54 years)	
Tumor site	Parotid gland	17 (26%)
	Submandibular gland	13 (20%)
	Sublingual gland	2 (3%)
	Oral cavity	15 (23%)
	Oropharynx	2 (3%)
	Nasopharynx	4 (6%)
	Paranasal sinuses	2 (3%)
	Larynx	2 (3%)
	Trachea	4 (6%)
	Ear	3 (5%)
	Esophagus	1 (2%)
Tumor	T1	22 (34%)
	T2	10 (15%)
	T3	13 (20%)
	T4	14 (22%)
	N/A	6 (9%)
Node	N0	56 (86%)
	N1	3 (5%)
	N/A	6 (9%)
Metastasis	M0	59 (91%)
	M1	0 (0%)
	N/A	6 (9%)
Stage	I	20 (31%)
	II	11 (17%)
	III	14 (22%)
	IV	13 (20%)
	N/A	6 (11%)
Perineural invasion	Yes	38 (28%)
	No	18 (58%)
	N/A	9 (14%)
Tumor‐infiltrating lymphocytes	< 10%	58 (89%)
	≥ 10%	7 (11%)
Tumor‐stroma ratio	Low	22 (34%)
	High	43 (66%)
Tumor budding	< 5 buds	22 (34%)
	≥ 5 buds	43 (66%)
Local recurrence	Yes	46 (71%)
	No	18 (28%)
	n/d	1 (1%)
Regional recurrence	Yes	4 (6%)
	No	60 (92%)
	N/A	1 (2%)
Distant recurrence	Yes	23 (35%)
	No	42 (65%)
Disease‐free survival	Yes	33 (51%)
	No	32 (49%)
Status	NED	27 (42%)
	AWD	3 (4.5%)
	DOD	22 (34%)
	DOC	11 (17%)
	N/A	2 (2.5%)

Abbreviations: AWD, alive with disease; DOC, dead on other cause; DOD, dead on disease; M, metastasis; N, node; N/A, not available; NED, no evidence of disease; T, tumor.

### 3.1 Histopathologic Examination

**FIGURE 1:**
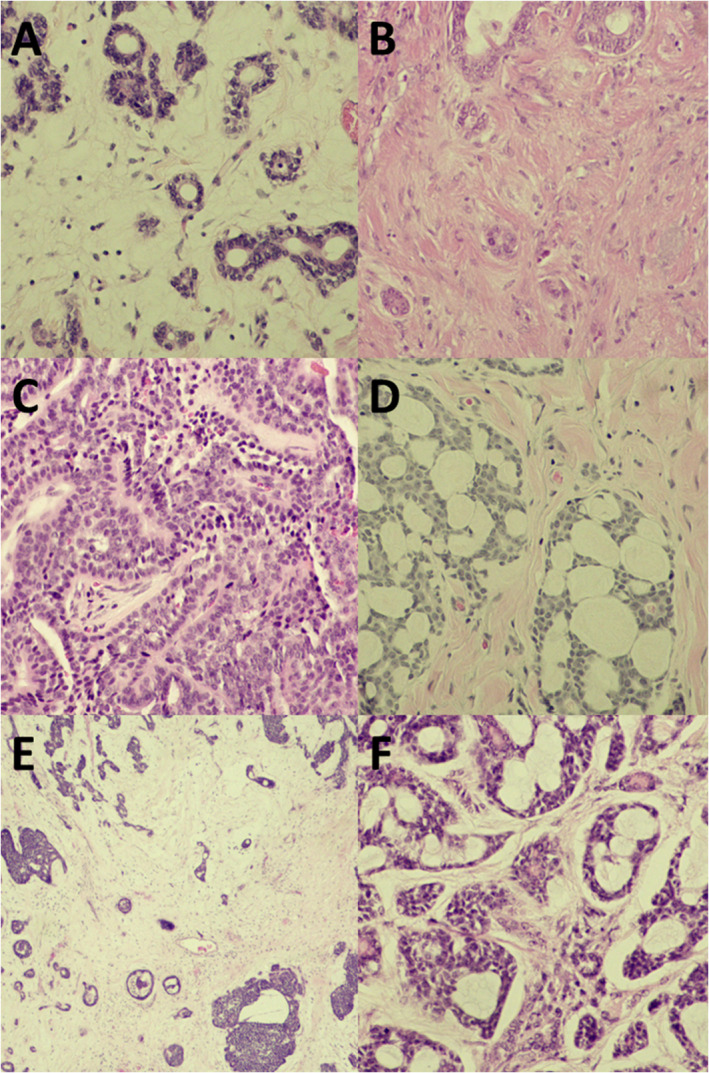
(A–F) Hematoxylin and eosin‐stained section of adenoid cystic carcinoma (×20). (A) Tumor budding in adenoid cystic carcinoma with more than 5 buds. (B) Tumor budding in adenoid cystic carcinoma with less than 5 buds. (C) Tumor‐infiltrating lymphocytes in the stroma of adenoid cystic carcinoma. Case with a high number of tumor infiltrating lymphocytes. (D) Adenoid cystic carcinoma with a low number of tumor infiltrating lymphocytes. (E) Stroma‐rich adenoid cystic carcinoma. (F) Stroma‐poor adenoid cystic carcinoma.

In analysis of tumor‐associated histopathologic features, 66.2% of ACCs showed a high intensity of tumor budding, i.e., 5 buds or more (Figure 1A,B). The prognostic value of tumor budding, TSR, and TILs was analyzed in the different growth patterns and no significant association was found (data not shown). Regarding stromal immune response, a large majority (89.2%) of the tumors had a low response with low infiltration of stromal TILs (Figure 1C,D). Moreover, the low infiltration of TILs was significantly associated with a higher tumor budding (*p* = 0.039, Fisher's exact test). A stroma‐rich stromal pattern was observed in most ACCs (66.2%). Tumors with stroma‐rich pattern as compared with stroma‐poor pattern (Figure 1E,F) were associated with more recurrences (*p* = 0.029) and more frequent distant metastases (*p* = 0.038).

**FIGURE 2:**
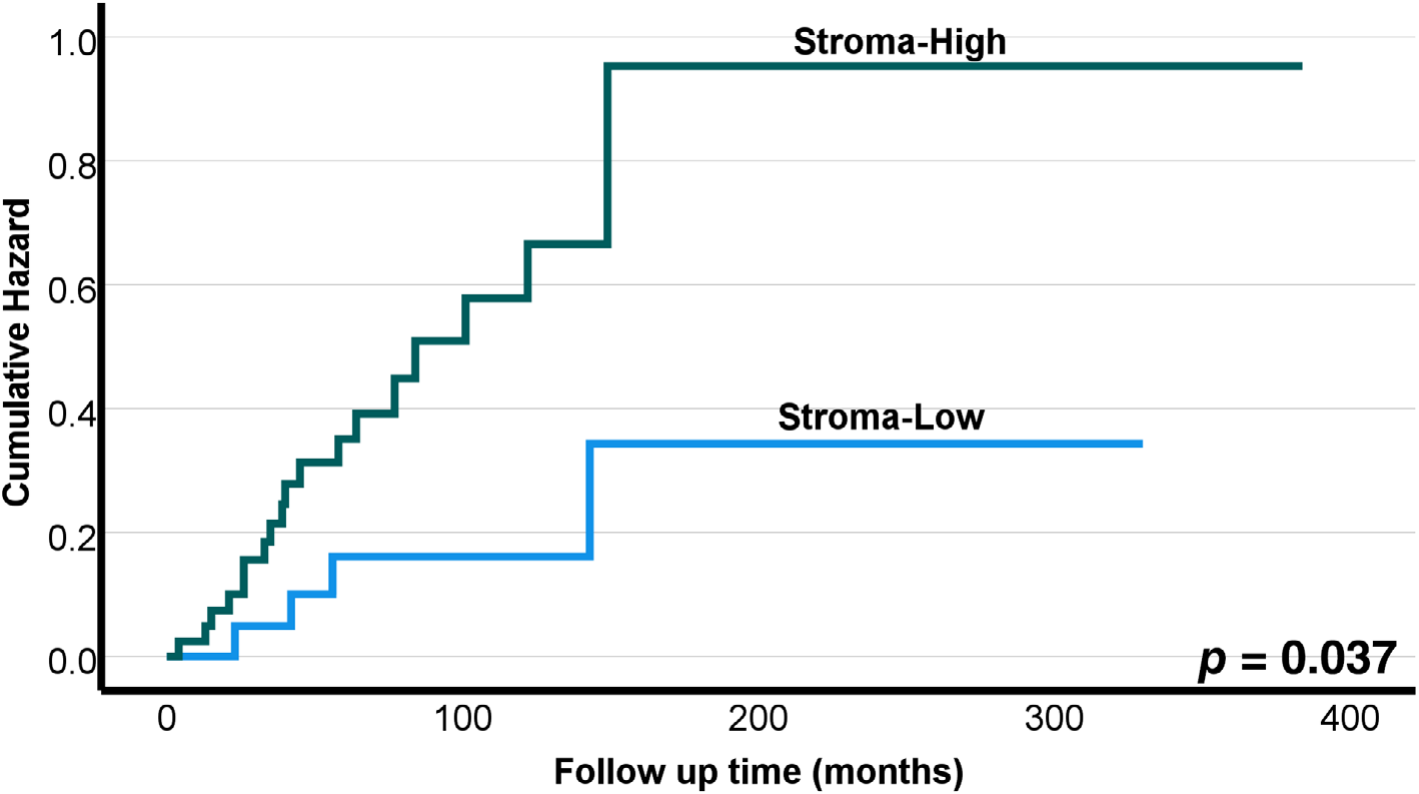
Disease‐specific survival with tumor‐stroma ratio in 65 patients treated for adenoid cystic carcinoma. There was a significant difference in the survival (*p* = 0.037) where stroma‐high (i.e., stroma‐rich) tumors associated with worse survival compared to stroma‐low tumors.

Tables 2, 3, and 4 present Cox regression analysis. In univariable analysis of Cox regression (Table 3), the stroma‐high (i.e., stroma‐rich) tumors were associated with worse DSS (HR 3.03, 95% CI 1.02–9.02, *p* = 0.046). This significant association between TSR and DSS was confirmed also by Kaplan–Meier survival curves (Figure 2). In OS and DFS analyses none of the studied histological features (TSR, TIL, and tumor budding) reached statistical significance. In multivariable analyses, only TSR reached a statistically significant value in predicting DSS with a HR of 3.76 (95% CI 1.10–12.83, *p* = 0.034).

**TABLE 2:** Overall survival analysis of 65 patients with adenoid cystic carcinoma.

Factor	Univariate analysis	Multivariate analysis		
		Model 1	Model 2	Model 3
	HR (95% CI)	HR (95% CI)	HR (95% CI)	HR (95% CI)
Tumor‐stroma ratio				
Low	1	1		
High	1.88 (0.87–4.05), *p* = 0.11	1.76 (0.75–4.15), *p* = 0.19		
Tumor‐infiltrating lymphocytes				
High (≥ 10%)	1		1	
Low (< 10%)	0.69 (0.26–1.79), *p* = 0.44		0.49 (0.17–1.39), *p* = 0.17	
Tumor budding				
Low (< 5 buds)	1			1
High (≥ 5 buds)	1.45 (1.44–3.04), *p* = 0.33			1.72 (0.69–4.29), *p* = 0.25
Age				
≤ 60	1	1	1	1
> 60	1.88 (0.94–3.78), *p* = 0.08	1.86 (0.80–4.32), *p* = 0.15	1.96 (0.85–4.51), *p* = 0.11	1.91 (0.83–4.43), *p* = 0.13
Gender				
Male	1	1	1	1
Female	0.37 (0.19–0.74), *p* = 0.005	0.33 (0.14–0.79), *p* = 0.01	0.31 (0.14–0.73), *p* = 0.007	0.35 (0.15–0.82), *p* = 0.02
Growth pattern				
Cribriform	1	1	1	1
Tubular	1.14 (0.26–4.99), *p* = 0.86	1.67 (0.35–8.06), *p* = 0.53	1.17 (0.24–5.69), *p* = 0.85	1.79 (0.36–8.89), *p* = 0.48
Solid	1.65 (0.71–3.82), *P* = 0.25	1.07 (0.40–2.82), *p* = 0.89	1.18 (0.46–3.03), *p* = 0.73	1.15 (0.45–2.96), *p* = 0.77
Cribriform and tubular	0.63 (0.25–1.60), *P* = 0.33	0.39 (0.12–1.30), *p* = 0.13	0.47 (0.14–1.54), *p* = 0.21	0.46 (0.14–1.47), *p* = 0.19
Perineural invasion				
None	1	1	1	1
Present	1.85 (0.75–4.57), *p* = 0.18	1.43 (0.52–3.93), *p* = 0.49	1.32 (0.49–3.59), *p* = 0.58	1.36 (0.49–3.72), *p* = 0.55

*Note*: Each one of the examined histopathologic features (i.e., tumor‐stroma ratio, tumor‐infiltrating lymphocytes and tumor budding) was entered into a multivariate model comprising age, gender, growth pattern and perineural invasion.

Abbreviations: CI, confidence interval; HR, hazard ratio.

**TABLE 3:** Disease‐specific survival analysis of 65 patients with adenoid cystic carcinoma.

Factor	Univariate analysis	Multivariate analysis		
		Model 1	Model 2	Model 3
	HR (95% CI)	HR (95% CI)	HR (95% CI)	HR (95% CI)
Tumor‐stroma ratio				
Low	1	1		
High	3.03 (1.02–9.02), *p* = 0.04	3.76 (1.10–12.83), *p* = 0.03		
Tumor‐infiltrating lymphocytes				
High (≥ 10%)	1		1	
Low (< 10%)	0.75 (0.22–2.56), *p* = 0.65		0.69 (0.17–2.74), *p* = 0.59	
Tumor budding				
Low (< 5 buds)	1			1
High (≥ 5 buds)	1.07 (0.45–2.55), *p* = 0.88			1.17 (0.41–3.38), *p* = 0.77
Age				
≤ 60	1	1	1	1
> 60	1.47 (0.61–3.54), *p* = 0.39	1.93 (0.65–5.71), *p* = 0.24	2.27 (0.80–6.39), *p* = 0.12	2.24 (0.78–6.42), *p* = 0.14
Gender				
Male	1	1	1	1
Female	0.35 (0.15–0.82), *p* = 0.02	0.16 (0.05–0.54), *p* = 0.003	0.19 (0.06–0.57), *p* = 0.003	0.19 (0.06–0.58), *p* = 0.004
Growth pattern				
Cribriform	1	1	1	1
Tubular	1.73 (0.38–7.86), *p* = 0.48	4.31 (0.73–25.36), *p* = 0.11	2.42 (0.42–13.95), *p* = 0.32	2.95 (0.52–16.89), *p* = 0.22
Solid	1.89 (0.69–5.13), *p* = 0.21	0.93 (0.28–3.17), *p* = 0.91	1.35 (0.43–4.22), *p* = 0.60	1.30 (0.42–4.09), *p* = 0.65
Cribriform and tubular	0.49 (0.14–1.75), *p* = 0.27	0.19 (0.04–1.11), *p* = 0.07	0.24 (0.04–1.34), *p* = 0.11	0.23 (0.04–1.24), *p* = 0.09
Perineural invasion				
None	1	1	1	1
Present	1.42 (0.51–3.97), *p* = 0.50	0.83 (0.24–2.83), *p* = 0.76	0.68 (0.19–2.37), *p* = 0.55	0.66 (0.19–2.31), *p* = 0.51

*Note*: Each one of the examined histopathologic features (i.e., tumor‐stroma ratio, tumor‐infiltrating lymphocytes and tumor budding) was entered into a multivariate model comprising age, gender, growth pattern and perineural invasion.

Abbreviations: CI, confidence interval; HR, hazard ratio.

**TABLE 4:** Disease‐free survival of 65 patients with adenoid cystic carcinoma.

Factor	Univariate analysis	Multivariate analysis		
		Model 1	Model 2	Model 3
	HR (95% CI)	HR (95% CI)	HR (95% CI)	HR (95% CI)
Tumor‐stroma ratio				
Low	1	1		
High	1.29 (0.47–3.51), *p* = 0.62	7.27 (0.92–57.43), *p* = 0.06		
Tumor‐infiltrating lymphocytes				
High (≥ 10%)	1		1	
Low (< 10%)	0.47 (0.13–1.66), *p* = 0.24		0.07 (0.003–1.62), *p* = 0.09	
Tumor budding				
Low (< 5 buds)	1			1
High (≥ 5 buds)	1.56 (0.55–4.45), *p* = 0.40			1.99 (0.36–11.06), *p* = 0.43
Age				
≤ 60	1	1	1	1
> 60	1.04 (0.38–2.85), *p* = 0.94	0.48 (0.09–2.62), *p* = 0.39	1.09 (0.11–10.97), *p* = 0.94	0.55 (0.09–3.04), *p* = 0.49
Gender				
Male	1	1	1	1
Female	0.43 (0.16–1.12), *p* = 0.08	0.16 (0.02–1.39), *p* = 0.09	0.19 (0.02–2.05), *p* = 0.17	0.52 (0.11–2.43), *p* = 0.41
Growth pattern				
Cribriform	1	1	1	1
Tubular	1.57 (0.19–13.33), *p* = 0.68	9.52 (0.44–205.71), *p* = 0.15	0.14 (0.001–34.05), *p* = 0.48	8.82 (0.37–211.99), *p* = 0.18
Solid	2.32 (0.57–9.34), *p* = 0.24	2.74 (0.44–17.31), *p* = 0.28	0.66 (0.05–8.40), *p* = 0.75	3.17 (0.39–25.28), *p* = 0.28
Cribriform and tubular	*p* = 0.97	*p* = 0.98	*p* = 0.98	*p* = 0.98
Perineural invasion				
None	1	1	1	1
Present	1.17 (0.32–4.25), *p* = 0.81	0.36 (0.06–2.13), *p* = 0.26	0.21 (0.03–1.53), *p* = 0.12	0.30 (0.05–1.88), *p* = 0.19

*Note*: Each one of the examined histopathologic features (i.e., tumor‐stroma ratio, tumor‐infiltrating lymphocytes and tumor budding) was entered into a multivariate model comprising age, gender, growth pattern and perineural invasion.

Abbreviations: CI, confidence interval; HR, hazard ratio.

## 4 Discussion

New approaches would be needed to better predict the behavior of ACC and aid in clinical decision making for ACC patients. To our knowledge, this is the first study on tumor‐ and stroma‐related histopathologic features (tumor budding, TSR, and TILs) in ACC. The profile of histopathological features of ACC in the current study is quite exceptional and generally opposite compared to other solid malignancies. Namely, in majority of ACCs budding was present, the number of TILs was low, and TSR was high which fit perfectly with the poor outcome of ACC. Low infiltration of TILs was associated with increased tumor budding. In addition, stroma‐rich tumors tended to recur more often, being in line with earlier studies demonstrating that stroma‐rich cancers have worse prognosis [12].

Tumor budding indicates low cohesion of tumor cells and it is a phenomenon related to epithelial‐mesenchymal‐transition [7]. Tumor budding has been studied in various malignancies including colorectal carcinoma and oral squamous cell carcinoma (SCC) [7]. In the current study, the intensity of tumor budding at ×20 magnification was graded into two groups using a cut‐off of 5 buds (low < 5 buds vs. high ≥ 5 buds). A similar protocol has been used widely in other studies [10]. We did not find correlation between tumor budding and prognosis. Lugli et al. points out that high tumor budding in solid cancers, such as colorectal cancer, is strongly associated with poor prognosis [7]. The predominantly solid growth pattern of ACC has been shown to be a sign of poor prognosis [22]. However, we did not find correlation between tumor budding and prognosis in the solid subtype. We showed that low infiltration of TILs was associated with a high number of tumor buds.

ACC is histologically comprised of myoepithelial and epithelial tumor cells, and it displays three histological growth patterns with dissimilar features of predominant histoarchitecture. In the tubular and cribriform types of ACCs, both of which have biphasic ductal structures, the occurrence of tumor budding may be restricted by the tumor architecture, and budding may take place less readily than in solid tumors of unicellular origin [23]. Furthermore, tumor budding in SCC and adenocarcinomas composed of a single cell type might represent a different phenomenon than in ACC. In view of the unique relentless clinical behavior of ACC we hypothesize that tumor spread in ACC may utilize different biological mechanisms than in colorectal carcinoma or oral SCC. Conceivably, only tumor budding in the solid growth pattern of ACC might be biologically comparable to budding in SCC.

In the current study, stroma‐rich ACCs of all three growth patterns recurred more frequently and showed poorer patient outcome. Similarly, in various other tumor types, including oral SCC, a high proportion of tumor‐associated stroma has been reported to correlate with poor prognosis [12, 24]. Thus, regarding TSR, ACC in its different forms behaves similarly to various other malignancies.

It is remarkable that most of the ACCs (89%) showed low infiltration of stromal TILs indicating a low immune response. The paucity of immune response suggests that ACC represents a salivary gland malignancy with low immunogenicity [25]. Additionally we found that low infiltration of TILs was associated with an increase in tumor budding, although we did not find correlation between tumor budding and prognosis. We hypothesize that in ACC low number of TILs might predict unfavorable response to immunological treatments. In head and neck SCC, immunohistochemistry for programmed death ligand‐1 (PD‐L1) has been widely used to predict the applicability of immune oncological treatments. Michaelides et al. reported PD‐L1 expression in ACC and demonstrated that tumor proportion score was zero in ACC [26] and predicted no benefit of PD‐L1 treatment. However, it remains to be studied whether macrophage function could be more important in immunity and tumor progression of ACC [26].

It may be seen as a limitation of this study that our patient material is scarce. However, we point out that our cases represent the benefit of single institution consecutive cases from a major university hospital in Finland and include all ACCs from minor and major salivary glands in this institution. HE staining was selected as it is used widely in routine histopathology and because of accessibility of such slides, thus providing good possibilities for tumor budding analysis in the context of this study. While cytokeratin staining offers enhanced specificity and sensitivity for detecting tumor buds, its use in pathology laboratories varies based on availability of immunohistochemistry services, possible research focus, or perceived need for higher sensitivity in complex cases.

In conclusion, stroma‐richness in ACC can be considered a potential marker of poor prognosis. Interestingly, low infiltration of TILs was prevalent in ACC, and it was associated with higher tumor budding. Our findings warrant further validation studies in larger cohorts of ACC.

## Author Contributions

**Aleksi Rytkönen:** methodology, formal analysis, investigation, writing – original draft. **Hanna K. Laine:** methodology, data curation, investigation, writing – review and editing. **Antti Mäkitie:** conceptualization, methodology, writing – review and editing, supervision, project administration, resources. **Caj Haglund:** methodology, writing – review and editing, project administration, resources. **Jaana Hagström:** conceptualization, methodology, formal analysis, investigation, writing – review and editing, supervision. **Alhadi Almangush:** methodology, formal analysis, investigation, writing – original draft. **Ilmo Leivo:** conceptualization, methodology, investigation, writing – review and editing.

## Ethics Statement

Appropriate Institutional Review Board (IRB) approval and waiver of consent per IRB guidelines were obtained for this study. The Helsinki University Hospital Research Ethics Board (HUS Regional Committee on Medical Research Ethics, Dnro 31/13/03/02/2010, 01 February 2010) approved the study design, which was retrospective and had no effect on the treatment of patients.

## Consent

The need for written informed consent was waived by the Finnish National Supervisory Authority for Welfare and Health (decision nro 425/05.01.00.06/2009 and 10 041/06.01.03.01/2012) due to the retrospective nature of the study.

## Conflicts of Interest

The authors declare no conflicts of interest.

## Data Availability

The data that support the findings of this study are available on request from the corresponding author. The data are not publicly available due to privacy or ethical restrictions.
